# Case Report: Efficacy and safety of dose-escalated Mazdutide, a GLP-1/GCGR dual agonist, in an adolescent with obesity, type 2 diabetes, and hyperuricemia

**DOI:** 10.3389/fendo.2025.1654506

**Published:** 2025-09-15

**Authors:** Wenfei Cheng, Zilong Chen, Puyu Li, Yingyu Zhang, Yujin Ma, Peng Liu, Hongwei Jiang

**Affiliations:** Henan Key Laboratory of Rare Diseases, Endocrinology and Metabolism Center, The First Affiliated Hospital, and College of Clinical Medicine of Henan University of Science and Technology, Luoyang, China

**Keywords:** adolescent obesity, type 2 diabetes mellitus, hyperuricemia, Mazdutide, GLP-1/GCGR dual agonist, adolescent treatment

## Abstract

**Objective:**

Mazdutide, a glucagon-like peptide-1/glucagon receptor (GLP - 1/GCGR) dual agonist, has shown marked efficacy in glycemic control, weight loss, and metabolic improvement in adults. However, data in adolescents remain limited. This report explores its therapeutic potential in an adolescent with obesity-related type 2 diabetes mellitus (T2DM) and hyperuricemia (HUA).

**Study design and methods:**

We report the case of a 15-year-old male patient diagnosed with obesity (BMI: 30.64 kg/m²), type 2 diabetes mellitus (HbA1c: 9.60%), and hyperuricemia (serum uric acid: 511 μmol/L). The patient underwent a dose-escalation regimen of Mazdutide (2 mg → 4 mg → 6 mg, administered subcutaneously once weekly) in combination with metformin and insulin to evaluate therapeutic efficacy and safety outcomes.

**Results:**

After 36 weeks, the patient showed significant improvement: weight decreased by 16.8 kg (18.89% BMI reduction), uric acid dropped by 37.00%, and HbA1c fell by 21.88%. No hypoglycemic episodes occurred. Lipid levels improved notably: triglycerides fell by 69.02%, total cholesterol by 13.65%, and LDL cholesterol by 17.27%. Hepatic steatosis resolved by week 14, as confirmed by ultrasound. No adverse events were reported, and benefits were sustained post-treatment.

**Conclusions:**

Mazdutide exhibited robust metabolic efficacy and good tolerability in an adolescent with obesity, T2DM, and HUA. It improved glycemic control, reduced weight and uric acid, reversed steatosis, and modulated lipid profiles. These findings support its potential as a comprehensive treatment for adolescent metabolic disorders.

## Graphical Abstract

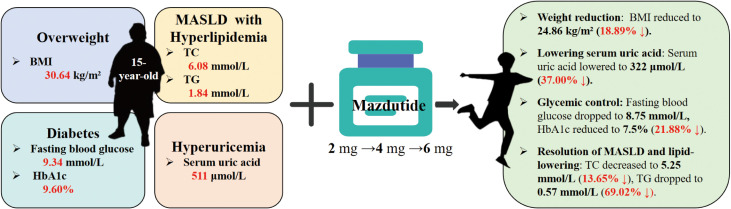

## Introduction

Adolescent metabolic syndrome has emerged as a significant public health concern, with the global prevalence of obesity, type 2 diabetes mellitus (T2DM), and hyperuricemia having doubled since 1990. Projections indicate that over 360 million adolescents will be affected by obesity by 2050, underscoring the critical need for effective management of metabolic comorbidities (1). Obesity not only impairs adolescent growth and development but also exerts long-term health consequences into adulthood, elevating the risk of cardiovascular disease, T2DM, hyperuricemia, and mortality (2). Concurrently, the prevalence of hyperuricemia among adolescents has risen due to lifestyle modifications, including increased consumption of purine-rich foods and sugar-sweetened beverages (3). Emerging evidence suggests that serum uric acid levels serve as a predictive biomarker for future essential hypertension and cardiovascular disease risk in pediatric and adolescent populations (4). Furthermore, the incidence of T2DM in adolescents has shown a marked increase in recent years. The extended subclinical phase of T2DM in demographic, coupled with insufficient awareness of disease risk among both patients and healthcare providers, contributes to a persistently high rate of underdiagnosis (5). These findings collectively emphasize the urgent need for more effective interventions to address the growing burden of metabolic syndrome in adolescents. Given the complex and multifaceted nature of this public health challenge, current medical approaches remain inadequate to meet the escalating demands.

In China, lifestyle modification is regarded as the first-line and foundational strategy for adolescents with type 2 diabetes; however, in practice, its effectiveness is often limited by poor adherence and marked interindividual variability in weight loss and metabolic improvement, which frequently necessitates the early initiation of pharmacologic therapy. Conventional therapeutic approaches, including metformin and insulin, demonstrate limited efficacy in adolescent populations and often fail to address multiple metabolic abnormalities (6). Although metabolic surgery has been shown to significantly improve metabolic indicators, some studies have raised concerns regarding its potential impact on pediatric growth and development, coupled with the inherent risks of surgical complications, necessitating careful consideration of its safety profile in children (7, 8). Furthermore, the application of existing uric acid-lowering agents in adolescents is constrained by safety concerns and ambiguous indications, highlighting the urgent need for novel therapeutic strategies (9).

Mazdutide, a novel GLP - 1/GCGR dual agonist, has demonstrated remarkable efficacy in glycemic control, weight reduction, and amelioration of metabolic syndrome in adult populations. Emerging evidence indicates that Mazdutide not only effectively reduces blood glucose levels and body weight but also significantly improves lipid profiles, blood pressure, and serum uric acid concentrations, thereby offering a promising therapeutic avenue for the comprehensive management of obesity, type 2 diabetes mellitus (T2DM), and hyperuricemia (10–14). Despite the well-documented efficacy and safety of Mazdutide in adults, its application in adolescent obesity and metabolic syndrome remains unexplored.

This case report investigates the therapeutic efficacy and safety profile of Mazdutide in the management of adolescent obesity complicated by metabolic syndrome, with the objective of contributing novel insights into treatment strategies for this condition. Through comprehensive documentation and systematic analysis of the clinical course in a 15-year-old male patient, this study aims to establish a foundation for potential future clinical applications and research endeavors in this domain.

## Case presentation

A 15-year-old male, born in November 2009, was identified with elevated fasting blood glucose levels (>6 mmol/L) during a routine physical examination in July 2020, despite the absence of overt symptoms such as polydipsia, polyuria, or xerostomia. Initial attempts to manage hyperglycemia through dietary modifications and physical activity proved ineffective. In September 2021, the patient presented to the hospital following a right forearm fracture, during which abdominal ultrasonography revealed hepatic steatosis. Subsequent glycemic monitoring demonstrated fasting blood glucose levels ranging from 7.6 to 8.9 mmol/L and postprandial glucose levels between 12.0 and 18.4 mmol/L. Glycemic monitoring included fasting and postprandial capillary glucose measured by home glucometer, and HbA1c assessed during hospital visits. Treatment with metformin hydrochloride (0.5 g three times daily) was initiated; however, neither glycemic control nor hepatic steatosis showed significant improvement. By April 2023, the patient returned due to persistent hyperglycemia, with fasting glucose levels recorded at 11.0 mmol/L (postprandial levels unmonitored). The therapeutic regimen was modified to include biphasic human insulin (30/70 mixture, 14 – 16 IU before breakfast and 14 – 18 IU before dinner) in combination with metformin (0.5 g three times daily), yet glycemic control remained suboptimal, as reflected by persistently elevated fasting plasma glucose and HbA1c levels. During a follow-up visit in March 2024, self-monitored fasting and postprandial glucose levels were 9.3 mmol/L and 18.0 mmol/L, respectively. The treatment protocol was further adjusted to include nightly subcutaneous administration of biphasic human insulin (30/70 mixture, 12 IU) alongside metformin hydrochloride (0.5 g in the morning and 0.75 g at noon). After six weeks of this regimen, no significant improvement in glycemic control was observed.

In March 2024, immediately prior to initiating Mazdutide therapy, A comprehensive clinical evaluation was performed on the patient, revealing the following findings: elevated fasting blood glucose at 10.18 mmol/L (reference range: 3.9 - 6.10 mmol/L) and HbA1c at 9.60% (reference value: <5.8%). Hyperuricemia was evident with serum uric acid levels of 511 μmol/L (reference value: <428 μmol/L), accompanied by dyslipidemia characterized by total cholesterol of 6.08 mmol/L (reference value: <6.0 mmol/L) and triglycerides of 1.84 mmol/L (reference value: <1.7 mmol/L). Hepatic ultrasonography demonstrated evidence of hepatic steatosis. The patient presented with congenital amblyopia and exhibited multiple metabolic risk factors, including a dietary pattern rich in high-sugar and high-fat foods, physical inactivity, obesity (weight: 87.5 kg, BMI: 30.64 kg/m²), and elevated blood pressure (138/83 mmHg). Physical examination revealed characteristic features of metabolic syndrome, including supracollarbone fat pads, buffalo hump, and acanthosis nigricans on the neck. A strong family history of diabetes was noted, with both parents and maternal grandparents affected, indicating a significant genetic predisposition. The patient was diagnosed with type 2 diabetes mellitus, obesity, hyperuricemia, and Metabolic dysfunction–associated steatotic liver disease (MASLD) with associated hyperlipidemia.

Given the complex interplay of multiple metabolic disorders, monotherapy demonstrated limited efficacy. Consequently, a comprehensive therapeutic regimen was initiated, consisting of weekly subcutaneous injections of Mazdutide 2mg, nocturnal administration of 30/70 Mixture Recombinant Human Insulin (12 IU), and oral Metformin Hydrochloride (0.5g in the morning, 0.75g at noon, and 0.5g in the evening). The detailed pharmacological interventions are further elaborated in the “Research Design and Methods” section.

## Research design and methods

Following informed consent, the treatment protocol was initiated with dose adjustment of Mazdutide, administered via subcutaneous injection once weekly (**Table 1**). The dose escalation phase comprised three stages: 2 mg during weeks 1 - 4, 4 mg during weeks 5 - 8, and 6 mg during weeks 9 - 12. The maintenance phase (weeks 13 - 36) continued at 6 mg, followed by a drug withdrawal observation period (weeks 37 - 42) during which Mazdutide was discontinued and metabolic parameters were monitored.

**Table 1:** Drug dosage adjustment plan.

Period	Timeframe	Dosage	Purpose
Titration Phase	Weeks 1 – 12	2-6mg	Establish tolerance
Maintenance Phase	Weeks 13 – 36	6mg	Maintain therapeutic efficacy
Discontinuation Phase	Weeks 37 – 42	0mg	Assess rebound risk

Concurrent therapeutic interventions included structured lifestyle modifications, consisting of a controlled diet—low in sugar, fat, and purines, with limited red meat and seafood intake, avoidance of alcohol and sugar-sweetened beverages, and increased consumption of vegetables, fruits, and low-fat dairy products—as well as regular aerobic exercise such as brisk walking or cycling for at least 150 minutes per week. Pharmacological therapy consisted of 12 IU of 30/70 Mixture Recombinant Human Insulin administered subcutaneously once nightly, combined with oral Metformin Hydrochloride (0.5 g morning, 0.75 g noon, and 0.5 g evening).

A comprehensive follow-up protocol was implemented, comprising both remote and in-person assessments. Weekly remote monitoring was conducted via telephone or digital platforms to track weight (measured using a calibrated home electronic scale with 0.1 kg precision), fasting blood glucose (assessed via portable glucometer), and adverse events (including nausea, vomiting, and diarrhea). In-person hospital evaluations were scheduled at 12, 14, 24, 28, 36, and 42 weeks (6 weeks post-withdrawal), with additional visits implemented as clinically indicated based on patient status.

## Results

**Figure 1:**
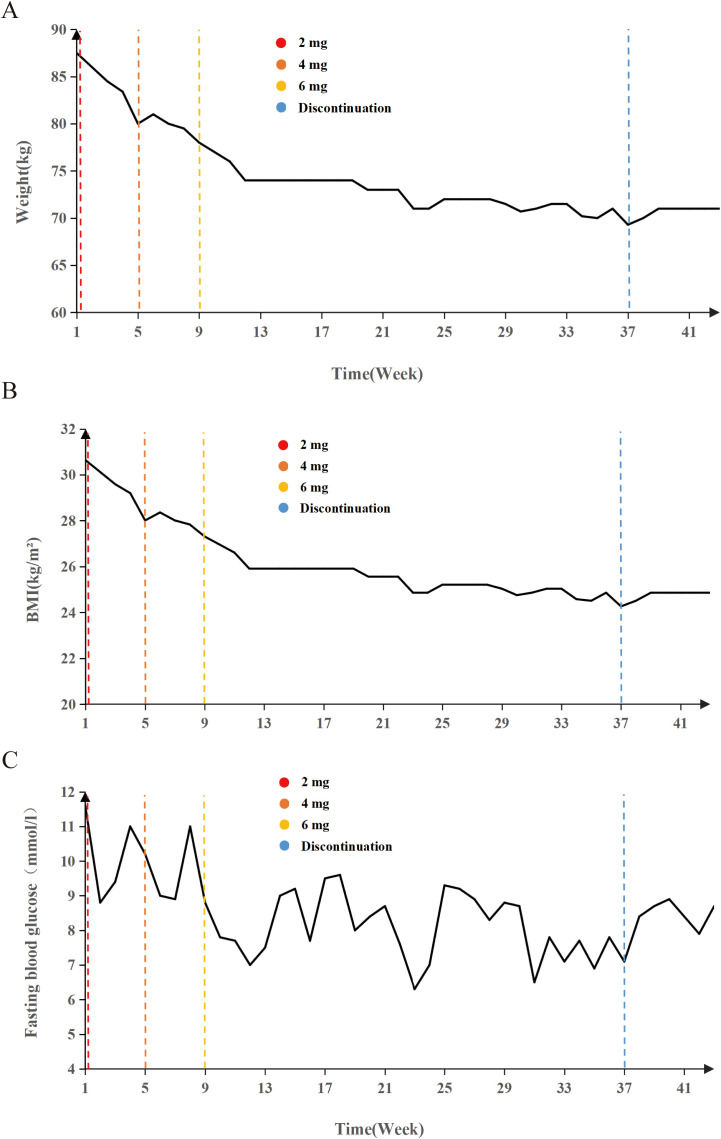
Longitudinal changes in weight, BMI, and fasting blood glucose during Mazdutide treatment. **(A)** Body weight (kg), **(B)** body mass index (BMI, kg/m²), and **(C)** fasting blood glucose (mmol/L) levels over a 42-week period in a 15-year-old adolescent treated with Mazdutide. Dose escalation was conducted in three phases: 2 mg (weeks 1 – 4), 4 mg (weeks 5 – 8), and 6 mg (weeks 9 – 36), followed by treatment discontinuation from week 37 onward. Colored dashed lines indicate timepoints of dose adjustment and discontinuation: red (2 mg), orange-red (4 mg), yellow (6 mg), and blue (treatment discontinuation).

**Figure 2:**
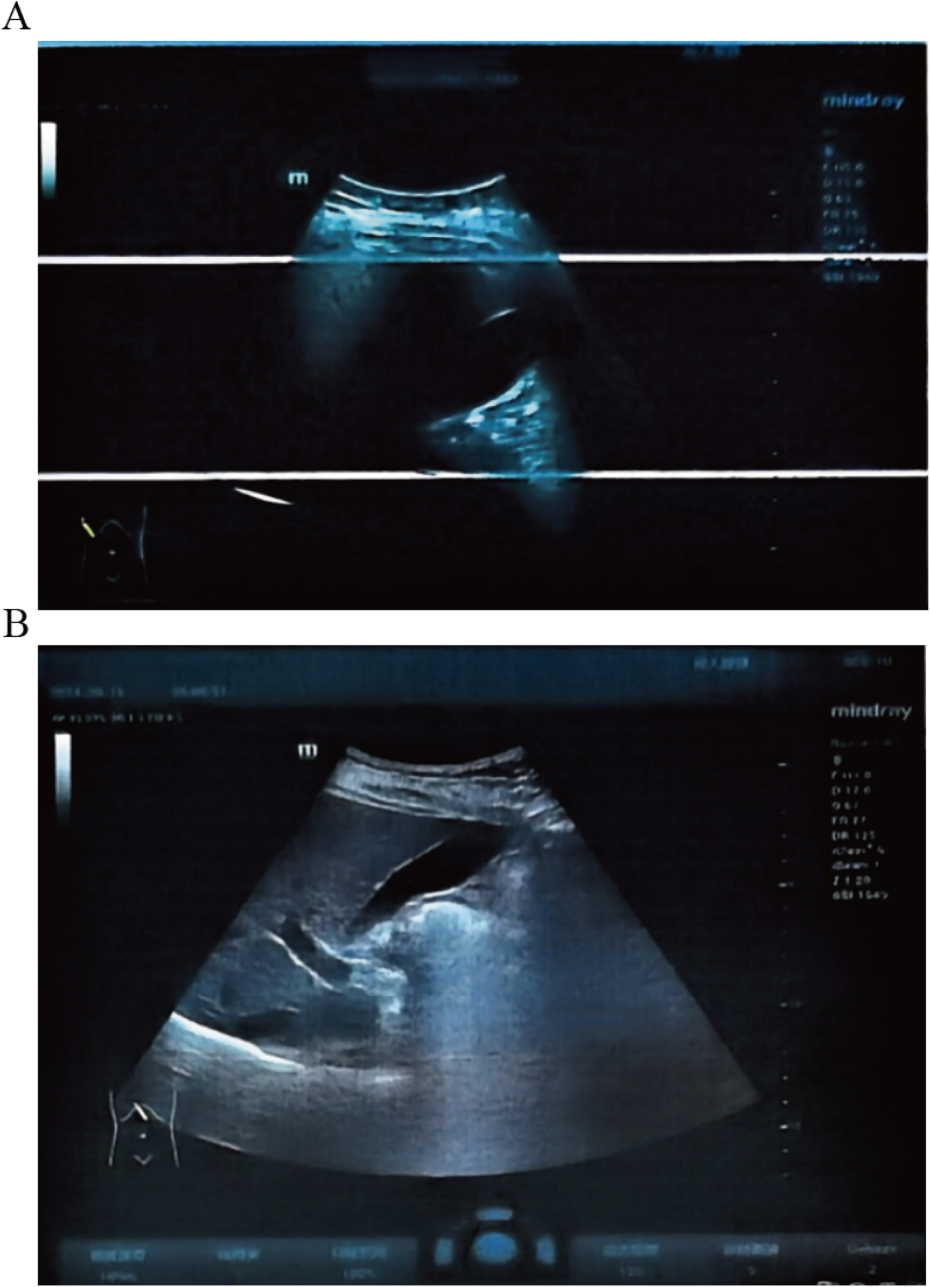
Liver ultrasound before and after Mazdutide treatment. **(A)** Baseline imaging shows hepatic steatosis with hepatomegaly and increased echogenicity. **(B)** Week 14 follow-up shows normalized liver size and echotexture, indicating resolution of steatosis.

Mazdutide resulted in significant metabolic improvements across multiple domains following a 36-week treatment period (**Table 2**). Body weight from 87.8 kg to 71.0 kg (Δ = -16.8 kg, -18.86%), accompanied by a decrease in BMI from 30.64 kg/m² to 24.86 kg/m² (Δ = -18.89%) (**Figures 1A, B**). Glycemic control parameters showed marked improvement, with fasting blood glucose decreasing from 11.0 mmol/L to 8.75 mmol/L (**Figure 1C**) and HbA1c levels reducing from 9.60% to 7.50% (Δ = -21.88%), with no reported episodes of hypoglycemia. Serum uric acid levels decreased from 511 μmol/L to 322 μmol/L (Δ = -37.00%), and the reduction was sustained 6 weeks after treatment discontinuation (322 μmol/L vs. baseline: 511 μmol/L). Lipid profiles also improved markedly: total cholesterol decreased from 6.08 mmol/L to 5.25 mmol/L (Δ = -13.65%); triglycerides showed a substantial reduction from 1.84 mmol/L to 0.57 mmol/L (Δ = -69.02%); high-density lipoprotein cholesterol increased from 1.05 mmol/L to 1.29 mmol/L; and low-density lipoprotein cholesterol decreased from 3.88 mmol/L to 3.21 mmol/L (Δ = -17.27%). Ultrasonography indicated resolution of hepatic steatosis: pretreatment imaging demonstrated hepatic steatosis characterized by hepatomegaly, fine enhanced parenchymal echoes, and absence of posterior acoustic attenuation (**Figure 2A**). Post-treatment imaging at 14 weeks showed normalization of hepatic size and morphology, with uniform parenchymal echotexture, improved sound transmission, and patent vascular flow, indicating complete resolution of hepatic steatosis (**Figure 2B**).

**Table 2:** Metabolic indicators.

Parameter	Baseline	Week 12	Week 14	Week 24	Week 28	Week 36	Week 42	% Change (Week 42 vs. Baseline)
Body weight(kg)	87.5	76	75	73	72.3	71	71	-18.86%
BMI (kg/m²)	30.64	26.61	26.25	25.56	25.3	24.85	24.85	-18.89%
HbA1c (%)	9.60	7.7	NA	7.8	6.9	8.8	7.5	-21.88%
Serum uric acid(μmol/L)	511	422	NA	406	388	372	322	-37.00%
TC (mmol/L)	6.08	5.3	4.69	3.61	NA	4.57	5.25	-13.65%
TG (mmol/L)	1.84	2.15	0.96	1.05	NA	0.97	0.57	-69.02%
HDL-C(mmol/L)	1.05	1.55	0.99	1.63	NA	1.47	1.29	22.86%
LDL-C(mmol/L)	3.88	3.24	3.01	2.16	NA	2.9	3.21	-17.27%
Abdominal Ultrasound	hepatic steatosis	NA	Normalized	NA	NA	NA	NA	NA

Body Weight and BMI: Reflect overall nutritional status and degree of obesity. In adults, BMI ≥24 kg/m² indicates overweight, and ≥28 kg/m² indicates obesity. During treatment, body weight decreased by 18.86% (from 87.5 kg to 71.0 kg), and BMI decreased by 18.89% (from 30.64 to 24.85 kg/m²).

Glycated Hemoglobin (HbA1c): Reflects average blood glucose levels over the past 2 – 3 months. The normal reference range is generally 4%–6%. During treatment, HbA1c showed some fluctuations and ultimately decreased by 21.88% at week 42 (from 9.60% to 7.50%).

Serum Uric Acid: Indicates purine metabolism and renal excretory function. The normal range is 149 – 416 μmol/L for males and 89 – 357 μmol/L for females. Levels progressively decreased during treatment, with a 37.00% reduction at week 42 (from 511 to 322 μmol/L).

TC: Used to evaluate lipid metabolism and cardiovascular risk.The normal range is 2.9 – 5.18 mmol/L. A 13.65% reduction was observed during treatment (from 6.08 to 5.25 mmol/L).

TG: Serve as a major form of energy storage and transport, derived from dietary intake or synthesized by the liver. The normal upper limit for males is <2.26 mmol/L. After a transient increase, levels declined significantly, showing a 69.02% reduction at week 42 (from 1.84 to 0.57 mmol/L).

HDL-C: Involved in reverse cholesterol transport, facilitating the removal of cholesterol from peripheral tissues to the liver for metabolism, and plays a protective role against atherosclerosis.The normal level for males is >1.04 mmol/L. HDL-C increased by 22.86% during treatment (from 1.05 to 1.29 mmol/L).

LDL-C: The primary atherogenic lipoprotein in plasma, which can deposit in the vascular wall and contribute to plaque formation and atherosclerosis progression. The normal range is <3.37 mmol/L. LDL-C decreased by 17.27% during treatment (from 3.88 to 3.21 mmol/L).

Abdominal Ultrasound: Used to assess abdominal organ structure and hepatic fat infiltration. Pre-treatment imaging suggested possible hepatic steatosis, while follow-up at week 14 showed normalization of liver size and morphology.

Mazdutide was well tolerated throughout the treatment period, with no adverse events or drug-related side effects reported. In particular, no episodes of hypoglycemia or hypotension occurred. The patient reported a high level of satisfaction, with no discomfort or treatment-related concerns. Although short-term therapeutic benefits were observed, longer-term studies are needed to evaluate the sustained safety of Mazdutide, including its impact on quality of life, psychological well-being, and long-term metabolic outcomes.

## Discussion

Mazdutide, a novel dual agonist targeting both the GLP - 1R and the GCGR, exerts not only the classical effects associated with GLP - 1R agonism—including enhanced insulin secretion, improved glycemic control, and appetite suppression—but also GCGR-mediated actions such as increased energy expenditure, promotion of hepatic fatty acid oxidation, and modulation of lipid metabolism. Notably, emerging clinical evidence suggests that Mazdutide may also significantly reduce serum uric acid levels, although the underlying mechanisms remain to be fully elucidated (15). While previous studies have established its comprehensive metabolic benefits in adults, this report represents the first documented application of Mazdutide in an adolescent with obesity and metabolic syndrome.

Given the growing global health burden of pediatric and adolescent obesity and its associated metabolic complications, this case provides pioneering clinical insight into the use of a GLP - 1R/GCGR dual agonist in this vulnerable population. The observed “quadruple therapeutic benefit”—comprising glycemic regulation, weight reduction, uric acid lowering, and reversal of hepatic steatosis—positions Mazdutide as a promising integrative therapeutic strategy for the comprehensive management of metabolic syndrome in adolescents.

The safety profile of Mazdutide in this case is particularly notable, especially given the critical developmental period of adolescence, during which drug safety and tolerability are of paramount concern. Throughout the treatment course, the patient experienced no adverse reactions, including no episodes of hypoglycemia or hypotension. These findings suggest that Mazdutide may exhibit a favorable safety and tolerability profile in adolescent populations, providing preliminary support for its potential broader application in the management of metabolic syndrome in this age group.

Although this study is limited to a single case with a relatively short follow-up period, the therapeutic efficacy observed has meaningful implications for addressing metabolic disorders in adolescents. Current treatment strategies for adolescent metabolic syndrome often face limitations, as conventional therapies typically target isolated disease components rather than offering comprehensive metabolic correction. Mazdutide, by contrast, appears capable of modulating multiple metabolic axes simultaneously—including glycemic control, weight reduction, uric acid lowering, and improvement in hepatic steatosis. This multi-faceted mechanism positions Mazdutide as a promising candidate for the integrative treatment of adolescent metabolic syndrome.

To better characterize the long-term efficacy and safety of Mazdutide in adolescents, future research should prioritize large-scale, longitudinal clinical trials. These studies should investigate optimal dosing strategies, treatment durations, and post-treatment follow-up protocols. Given the rising global prevalence of metabolic syndrome in adolescents, the clinical potential of Mazdutide offers a compelling avenue for therapeutic intervention. Continued investigation is warranted to establish robust evidence supporting its use in this vulnerable population.

## Limitations

This report describes a single patient with a relatively short follow-up period, the findings should be viewed as preliminary. Moreover, the therapeutic effects observed cannot be solely attributed to Mazdutide, since the patient also received concurrent metformin and insulin therapy. These observations nevertheless highlight the potential role of Mazdutide within a comprehensive treatment strategy and underscore the need for larger, longer-term studies.

## Conclusion

This case report highlights the therapeutic outcomes of Mazdutide in a 15-year-old adolescent with obesity-related type 2 diabetes mellitus (T2DM) and hyperuricemia. The findings demonstrate Mazdutide’s multifaceted efficacy, including improvements in glycemic control, weight reduction, lipid metabolism, hepatic steatosis, and serum uric acid levels. These results point to its potential as an integrated treatment option for pediatric metabolic disorders. However, the single-case design and short follow-up period limit the generalizability of these findings. Further large-scale, long-term clinical trials are needed to validate the safety and efficacy of Mazdutide and to guide evidence-based treatment strategies in adolescent populations.

## Data Availability

The original contributions presented in the study are included in the article/supplementary material. Further inquiries can be directed to the corresponding author.
